# Management of “De Novo” Carpal Tunnel Syndrome in Pregnancy: A Narrative Review

**DOI:** 10.3390/jpm14030240

**Published:** 2024-02-23

**Authors:** Maria-Cristina Cîmpeanu, Nadinne Roman, Simona Grigorescu, Ovidiu-Dan Grigorescu, Roxana Steliana Miclăuș

**Affiliations:** Faculty of Medicine, Transilvania University of Brașov, 500036 Brașov, Romania

**Keywords:** pregnancy-related carpal tunnel syndrome, preventive approach, personalized therapy, surgical decision, quality of life

## Abstract

Carpal tunnel syndrome is a common entrapment neuropathy that can occur in the upper limbs during pregnancy. However, it is often undervalued and underdiagnosed because the symptoms can be mistaken for regular changes during pregnancy. Delay or absence of diagnosis and treatment can lead to permanent nerve damage, which can affect a woman’s quality of life during and after pregnancy. Although the treatment protocols are well established for carpal tunnel syndrome in patients from the general population, there is a different situation among pregnant and postpartum women that requires a preventive and personalized approach to manage this pathology. Unfortunately, the range of available treatment options is limited due to the pregnant woman’s physiological status and influenced by the high possibility of the complete resolution of this pathology in the postpartum period without any treatment. To limit the possibility of unfavorable evolution of this syndrome during pregnancy, an early diagnosis and personalized approach are mandatory in each case involving a multidisciplinary team of general practitioners, obstetricians, hand surgeons, rehabilitation physicians, physiotherapists, and occupational therapists.

## 1. Introduction

Carpal tunnel syndrome (CTS) is the most frequent peripheral nerve entrapment syndromes that affects the upper limbs, particularly in females, with a ratio of 3:1 to 10:1. The “de novo” occurrence of CTS during pregnancy, also known as pregnancy-related carpal tunnel syndrome (PRCTS), ranges from 0.23% to 70% of cases. CTS is most commonly observed in the third trimester (63%), followed by 26% in the second and 11% in the first [1,2,3,4,5,6]. Unlike classical cases, where the treatment is determined by the patient’s clinical status (a wide range of surgical and non-surgical therapeutic approaches being available), the spectrum of therapeutic options (invasive and pharmaceutical) during pregnancy is limited due to the physiological status of the pregnant woman. This limitation is also based on the idea of a possible existing transient neuropathy in the context of pregnancy-specific changes. The CTS clinical picture may persist for up to approximately three years post-birth, so choosing an optimal therapeutic approach, even postpartum, becomes a challenge [2,3,7].

The main objective of this study was to assess the current therapeutic measures for pregnancy-related carpal tunnel syndrome (PRCTS) mentioned in the pertinent literature and to highlight the manner in which they could be managed in a predictive way. Additionally, we aimed to emphasize the benefits of early medical rehabilitation measures that can be applied in a preventive and personalized manner for cases where carpal tunnel syndrome develops during pregnancy. We also assess the impact of rehabilitation and complementary measures on the decision to perform the surgical procedure only after childbirth.

## 2. Clinical Findings and Diagnosis

The clinical picture of PRCTS is primarily determined by hormonal changes, postural modifications, weight gain and fluid retention. Increased serum levels of estrogen, aldosterone, cortisol, prolactin, and glucose usually cause these hormonal changes. The fluid retention causes edema, mainly determined by decreased venous return.

Several musculoskeletal system changes occur during pregnancy, with a significant increase in relaxin hormone production starting from the 18th week. The high levels of relaxin can also lead to the relaxation of the carpal ligament, followed by inflammation of the median nerve, flattening, increasing size, and impingement [1,7,8,9].

These physiological changes are typically seen in the last trimester of pregnancy and are reversible after birth. However, there is a possibility that some of these changes related to the PRCTS become definitive after delivery and develop into a chronic carpal tunnel syndrome similar to lower back pain syndrome, de Quervain’s syndrome, patellar chondromalacia, femoral head osteonecrosis, lower-limb cramps, and transient osteoporosis [1,7,8,9].

The clinical status during pregnancy is similar to that occurring in the general population. The pregnant woman can experience hand and radiocarpal joint pain, which usually affects the hands bilaterally and worsens when using both hands. Paresthesia, or a tingling sensation, can also occur, indicating nerve damage due to compression and ischemia. Pain interferes with daily activities, occurring both day and night. Nocturnal pain can cause significant disruptions in sleep quality, especially in the third trimester, potentially leading to physical and psychological strain on the mother and potentially lead to premature birth. The physical examination may reveal sensory disorders along the median nerve distribution, where variations in the nerve’s anatomy can cause impaired grip strength and prehension deficits, accompanied by hypotrophy/atrophy of the thenar muscles, which can be observed distal to the compression site [2,3,8,9,10,11,12].

The diagnosis is established, similarly to the general population, through medical history and physical examination, completed by electrophysiological studies. Both medical history and physical examination target the same parameters, as do the most common neurodynamic tests of Tinel and Phalen. Regarding electromyographic studies, it is important to note that these are not usually recommended during pregnancy due to the pain associated with nerve stimulation. Appropriate alternatives for clinical testing are the classic ultrasound examination or the recent high-frequency ultrasonography (HF-USG) method. Sonography has many advantages (non-invasive, high availability, low cost, no pain, shorter examination) and allows us to establish important findings on CTS, such as increased cross-sectional area of the median nerve, the hypoechoic pattern of the median nerve due to neural edema, and the hourglass appearance of the median nerve due to compression beneath the transverse carpal ligament. In addition, by using the Doppler examination, it is possible to also evaluate the vascularity of the forearm and hand. Certain anatomical variations of the median nerve can to be found and assessed [5,13,14]. A new diagnostic method, first described by Andrew Taylor Still, is represented by “myofascial release” based on the possibility to obtain persistent palpable feedback during attempts to release myofascial tissues. It requires a good knowledge of the upper-limb anatomy [2]. Once the final diagnosis is undoubtedly established, ruling out all other possible differential diagnoses, it is mandatory to determine the severity of the syndrome in order to adopt an optimal personalized therapeutic approach. Since, as mentioned earlier, electrophysiological studies are not universally recommended during pregnancy, the severity of symptoms and the functional status of the hand will be highlighted through other methods, such as the Boston Carpal Tunnel Questionnaire. This questionnaire evaluates two scales:

❖ SSS (symptom severity scale): this scale assesses the severity of symptoms concerning the timing, frequency, and severity of symptom occurrence.
❖ FSS (functional status scale): this scale is more complex, including the assessment of each symptom intensity related to the timing of rest (mainly nocturnal) or daytime activities.

The prognosis of this pathological status is usually favorable, with symptoms being almost entirely reversible after childbirth. The clinical picture can improve in the first weeks postpartum until about three years after birth, depending on the return to regular hormonal status, the decrease in fluid retention, and the return to normal use of the affected upper limb. Regression of the disease is faster in primiparous women. Postpartum disease persistence has been more frequently observed when its onset occurred in the first two trimesters of pregnancy and if it was associated with depression-like disorders. The changes in the physiological constants may persist for a longer, more extended period. The postpartum reversibility of CTS is a crucial aspect that complicates the choice of treatment type, especially decision-making regarding the initiation of surgical measures and the optimal timing for these [7,8,15,16,17].

## 3. Therapeutic Approaches

The treatment of carpal tunnel syndrome (CTS) in the general population is often closely correlated with the severity of the clinical status. Therefore, the therapeutic approach can be conservative, involving patient education, splinting/immobilization, vitamin therapy, non-steroidal anti-inflammatory drugs (NSAIDs), oral and injectable corticosteroids, and job changeset, or it can be a surgical one using the decompression approach.

It is well known that in pregnant women, various medical options, both conservative and surgical, can disrupt the physiological state and the normal progression of both the mother and the fetus. Thus, during pregnancy, the traditional therapeutic approach to CTS will undergo modifications and therapeutic adaptations to improve the clinical picture without affecting the pregnancy and/or the mother. The decision regarding the optimal timing and type of therapeutic measures must be personalized, guided by the severity level of the clinical status, and in correlation with the patient’s preferences. It can often become a genuine medical challenge involving a complex team: general physician, obstetric surgeon, hand surgeon, rehabilitation physicians and specialized therapists.

The increased frequency of pregnancy-related carpal tunnel syndrome (PRCTS) occurrence and the potential impact of symptoms on the patient (both during and after pregnancy) have sparked scientific interest in various studies. These studies mainly aimed to establish the most appropriate therapeutic measures to meet the aforementioned objectives. The therapeutic measures described in the pertinent literature for PRCTS that occurs during pregnancy include:

❖ Conservative management.
❖ Surgical management.

### 3.1. Conservative Management

#### 3.1.1. Patient Education

This is the first measure that should be adopted, involving early explanation to the pregnant patient of the disease pathophysiology, its dynamics and evolution, with particular considerations related to pregnancy and the postpartum period. It aims to present to every patient the preventive and therapeutic methods as part of personalized management of PRCTS, considered self-management techniques.

In this category are included:

▪ Sleeping position: avoiding excessive flexion movements during sleep.
▪ Awareness of trigger activities: understanding those that may trigger or worsen symptoms and avoiding or limiting them.
▪ Professional activity changes: making changes in professional activities to minimize strain.
▪ Home physical therapy programs: implementing home-based physical therapy programs, including stretching exercises, occupational therapy, nerve gliding exercises, yoga, Pilates, etc. [9,18,19].

#### 3.1.2. Work Task Changesets

These represent a series of ergonomic modifications in the workplace, primarily focusing on hand positioning during activities. They apply to mild/moderate forms of the disease or as a preventive measure. These modifications include adapting tools/equipment to optimize hand positioning, avoiding prolonged hyperflexion at the radiocarpal level, steering clear of lifting heavy weights, and organizing repetitive activities to include breaks or changes in position, etc. [20].

#### 3.1.3. Splint Immobilization

This approach is based on the idea that pressure within the carpal tunnel varies directly with positional changes, especially flexion and extension. The lowest pressure is found in the neutral position of the radiocarpal joint. Adopting this position can improve hemodynamic parameters, reduce edema, decrease nerve compression and friction, and alleviate pain.

Immobilization is one of the conservative therapeutic methods, applied either as monotherapy or more commonly in combination with other therapies. It is a simple, safe, cost-effective method effective from the early stages and beneficial in moderate stages before the onset of paresthesia. Correct immobilization can solve up to 80% of symptoms in mild-to-moderate cases of pregnancy-related carpal tunnel syndrome (PRCTS).

Choosing the correct type of orthosis is crucial, considering not only the material used but also the manufacturing type (customized/standard) and the positioning. For example, orthoses with a slight degree of extension, which can increase the pressure within the carpal tunnel and exacerbate symptoms, must be avoided [20].

Unlike classic cases of CTS, where the recommended wearing period for orthoses is well established (approximately six weeks), the recommended duration during pregnancy is unclear. Given the possibility of spontaneous resolution of symptoms postpartum, it has not been a relevant study objective.

Regarding the optimal wearing time, most hand therapists recommend intermittent use, especially during the night and activities that strain the radiocarpal joint or could exacerbate symptoms. In cases with persistent daytime symptoms and deficient compliance with night splinting, a particular type of orthosis was conceived: DayTimer splint. This type of splint seems to be the most suitable and has some remarkable features (adjustable loops) that permit only minimal interference with daily activities [20].

The involvement of a highly specialized hand therapist should be mandatory, with the responsibility to adjust the hand position continuously, establish the wearing schedule of the orthosis, and monitor the evolution of symptoms. Also, the therapist must identify as soon as possible the moment when the patient needs to be examined by the hand surgeon to decide if the surgical approach becomes mandatory [2,20,21,22].

#### 3.1.4. Physical Therapy

Physical therapy consists of therapeutic methods based on a principle/philosophy that highlights the concept that physical exercises (in their various forms) can lead to the regaining and enhancement of flexibility, endurance, strength, and range of motion and decrease in physical and psychological discomfort.

Unlike other therapeutic methods, it is essential to note that physical activity requires proper patient compliance (meaning the understanding of the biological program necessity and adherence to it and the correctly application at home of the learned exercise programs). The therapy will be personalized, agreed upon with the rehabilitation physician and therapist, and designed and supervised throughout each stage by them [5].

Numerous physical therapy techniques are described in the literature, applied as monotherapy or combined. Alongside well-known programs such as physical therapy, stretching, Pilates, and occupational therapy, several specific neuromuscular pathology techniques have proven helpful in PRCTS.

#### 3.1.5. Nerve and Tendon Gliding Exercises

These exercises are based on the idea (promoted by Wilgis and Murphy) that some cases of median nerve compression occur due to adhesion between the nerve epineural sheath and adjacent structures, causing thickening and limiting the nerve’s movement within the canal, subsequently reducing its perfusion and function [20,22].

**Figure 1:**
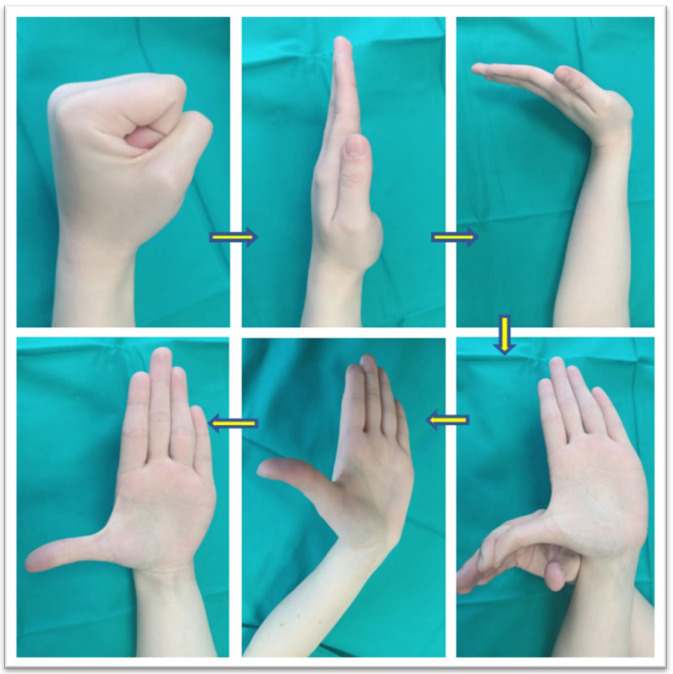
Median nerve gliding exercises: finger movements sequence; adapted from the therapeutic exercises program for carpal tunnel syndrome (American Academy of Orthopedic Surgeons).

Mobilization techniques of the flexor tendons lead to direct mobilization (gliding) of the nerve, facilitating venous return, significantly reducing local edema, decreasing pressure within the carpal tunnel, and subsequently improving the axonal transport and nerve conduction. One of the advantages of this technique is the possibility of performing these exercises at home after appropriate patient training. Special attention must be given to the rhythm and intensity of these techniques to avoid exacerbating symptoms through overuse [21]. Figure 1 presents one possible movement sequences for median nerve gliding adapted from the therapeutic exercise program for carpal tunnel syndrome designed by the American Academy of Orthopedic Surgeons.

The benefits of association with other therapeutic interventions (usually with splinting) and the efficacy of these exercises are widely debated topics [22,23,24,25,26,27]. Some studies have shown that mobilization techniques of the flexor tendons can have beneficial results in terms of the need for surgical intervention. For instance, one study revealed that among 200 hands examined, only 43% of those who underwent exercises required surgery compared to 71% who needed surgery without these exercises. On the other hand, there are studies (Akalin et al., Page et al.) suggesting that these exercises may not be beneficial, are inferior to immobilization, and in certain circumstances may even worsen symptoms [23,25,26,27].

The applicability of these exercises, targeting selected cases with continuous monitoring, is still debatable. The exercise program can be stopped in the absence or improvement of symptoms. To identify cases for whom the gliding exercises program is suitable, dynamic ultrasound imaging could be a helpful method. Dynamic ultrasound imaging can be easily performed in the clinical setting to assess the normal or abnormal gliding of the median nerve and flexor tendons within the carpal tunnel during different passive and active movements. Additionally, it can be used during the follow-up phase after conservative treatments [28,29].

#### 3.1.6. Neuromuscular Reeducation

This procedure involves a series of exercises (which can be assisted manually or mechanically) with the primary objective to increase muscle strength and improve the neuromotor response, proprioception, kinesthetic sense, balance, coordination, and posture [30]. Given the fact that in CTS, damage to the sensory functions of the median nerve is more common than the motor functions, maintaining and restoring sensations associated with the hand (such as touch, pain, or pressure) should be considered highly important and must be prioritized [30].

#### 3.1.7. The Use of Kinesio Tape (Kinesio Taping)

This represents a modern stretching method that has proven to positively affect numerous body systems (circulatory, lymphatic, neuromuscular, articular), both in healthy individuals and in various systemic dysfunctions. During pregnancy, the main benefits of applying Kinesio tape are increased mobility and stability, reduction in edema, decrease in muscle tension, and activation of pain modulation pathways. Regarding the direct benefits of Kinesio taping in carpal tunnel syndrome therapy, the main mechanism is represented by a reduction in fasciae hypertension, with subsequent space enlargement, reduction local inflammation, and decrease in the transverse cross-sectional area of the median nerve [31].

Additionally, in a recent study, Movaghar et al. highlighted the positive effects of using Kinesio tape in combination with cupping therapy on a group of 30 pregnant women with CTS. Both methods are considered non-invasive, safe, and financially accessible for patients with CTS [31].

#### 3.1.8. “Myofascial Release” Therapy

Myofascial relaxation techniques focus on identifying and releasing the myofascial trigger points, which are located in a taut band of skeletal muscle defining the so-called contraction knot. Likewise, fascial nociceptors are also involved in myofascial pain. Some of the histopathological characteristics of the myofascial syndrome (carpal tunnel-related), like synovial tissue hypertrophy, microcirculation injuries, and nerve connective tissue alterations, can emphasize the role of the myofascial release techniques in the management of PRCTS [32].

This therapy is considered both a diagnostic and therapeutic procedure, involving application by a specialized therapist (using direct and indirect methods) or even via a self-applied myofascial release method. The most common manual techniques used in the management of fascial pain are deep manual massage, soft-tissue mobilization, and compression of the myofascial trigger point [33].

Hamoda et al. conducted a study on 30 pregnant women randomly divided into two equal groups: a control group and those who received three daily sessions of myofascial therapy for four weeks. Additionally, women in both groups wore orthoses overnight. The results were measured through clinical reactivity and electrophysiological tests. In both groups, women described a clinical improvement in paresthesia attributed to wearing the orthosis. In the group that received myofascial therapy, a significant resolution of paresthesia, pain intensity, muscle weakness sensation, increased muscle strength, and grip, as well as an electrophysiological improvement (DML), were observed, which was absent in the control group [2].

#### 3.1.9. Mechanical Traction

This involves hand traction through a dynamic and pneumatic device aimed at progressively increasing the carpal tunnel’s dimension through progressive stretching along the transverse carpal ligament.

Studies have demonstrated a reduction in symptomatology for at least seven months in the general population. For pregnant women, data are limited but promising [3,9].

#### 3.1.10. Complementary Methods

Complementary methods accepted during pregnancy have been described, including yoga and acupuncture. Garfinkel and colleagues recommend yoga therapy to improve grip strength and decrease pain levels. Studies have not highlighted electrophysiological improvements, but rather improvements in posture and overall condition [20].

#### 3.1.11. Pharmacological Treatment

- Symptomatic treatment

During pregnancy, the decision to initiate a therapeutic scheme involving pharmacological substances must be balanced, considering the absolute need for medication administration and the potential side effects on both the fetus and the mother.

Pharmacological management of CTS addresses inflammatory, acute, or exacerbated phases unresponsive to conservative therapeutic means. These include: (a) non-steroidal anti-inflammatory drugs, used cautiously, especially after the 30th week; ibuprofen has shown satisfactory results, unlike other preparations; and (b) analgesics: acetaminophen as first line for mild-to-moderate pain. Aspirin can also be used, but preferably in the first two trimesters of pregnancy, with the dangers outweighing the benefits in its late stages (risk of postpartum hemorrhage and neonatal bleeding) [6,20]. Although the current data do not confirm serious evidence of teratogenic effects, aspirin remains the only NSAID with statistical significance regarding the occurrence of cryptorchidism and gastroschisis [34,35].

b. Injectable corticosteroids

Considered by most authors as ineffective in the long term and for this reason to be avoided in the general population, corticosteroids were found to be effective during pregnancy, resulting in a low recurrence rate in the short term and without harmful effects on the fetus or mother [5]. The most frequent indication related to corticosteroid therapy is acute CTS, developed in the third trimester. This therapy alleviates the symptoms for a short period, allowing an unaffected delivery and after-birth period. The advantage of this approach is the occurrence of spontaneous remission or reduction in CTS symptoms. The therapy can be applied in mild and moderate cases with persistent symptoms unresponsive to other treatments. Nowadays, the most accurate injection technique is applied by using ultrasound guidance to prevent any procedural-related complications, such as paresthesia or tendon damage. For corticosteroids used during pregnancy, it seems that the best option is dexamethasone acetate, being safe and ensuring reasonable control of symptom intensity during pregnancy [5,20,21,36,37].

This procedure could also be utilized as a discriminatory test for the pain caused by CTS or the cervical spine.

c. Vitamin Therapy

The administration of vitamin B12 is based on the theory of the involvement of its deficiency in the etiopathogenesis of CTS and its potential nerve-healing properties [20].

Alpha-lipoic acid is an important antioxidant and detoxifier that modulates numerous inflammatory pathways. According to studies, continuous administration of CTS for over three months has led to a significant improvement in symptoms and increased nerve conduction velocity. Regarding its administration during pregnancy, there are experimental animal studies suggesting significant advantages of administration during pregnancy and no contraindications [20,37].

The role of vitamin D in the treatment of metabolic syndrome, which could occur in pregnancy, causing a negative impact on peripheral nerve metabolism (including the median nerve) is still being investigated [38].

#### 3.1.12. Modalities

The available data on the safety and role of applying physiotherapy procedures during pregnancy are limited. Among the physiotherapy procedures accepted for use in this period, although not unanimously, are the following.

(a) High-frequency laser therapy has a specific role in reducing the local inflammatory process and pain relief. We found a single study regarding this therapy during pregnancy, including 54 pregnant with mild and moderate forms of CTS, in which Ashour et al. demonstrated an improvement in clinical symptoms and nerve conduction parameters with high-frequency laser therapy associated with standard physical therapy instead of standard physical therapy only [39].
(b) Ultrasound therapy and the deep-pulsed form can decrease pain intensity and paresthesia symptoms and even improve nerve conduction parameters. In PRCTS, it was applied to a small number of patients, mild/moderate cases, with contradictory results (authors like Ebenbichler support its benefit, confirmed both clinically and electro-physiologically, while others like Oztas consider this therapy to have no benefits, especially in cases of already existing nerve damage) [21,40].
(c) Iontophoresis is a process of transdermal drug delivery by use of the voltage gradient of the skin, which uses electrically charged ions to deliver topical agents applied on the skin. In pregnancy, the most suitable drug for carpal tunnel syndrome could be dexamethasone, but there is a lack of studies regarding the safety of this procedure. Moreover, there are some recommendations against the use of this procedure or at least a warning of using it with extreme caution [41,42].
(d) Phonophoresis uses ultrasound to facilitate the delivery or absorption of topical agents (usually corticosteroids). In PRCTS, phonophoresis seems to be a safe, non-invasive method, ensuring clinical improvements (by reducing local inflammation and pain and increasing the grip strength) and also of electrophysiological parameters [43].
(e) Hot-sand bath therapy is a newer method proposed for use in PRCTS. The only research we have highlighted (on a single patient) led to uncertain results (even if the authors considered that hot-sand bath therapy could decrease the intensity of pain and the related symptoms, also improving hand function) [44].

### 3.2. Surgical Management

The predictability of the need for surgical intervention during pregnancy is limited, due to the varying degrees of reversibility of postpartum pathology, occurring at different times from birth up to three years. Therefore, surgical treatment is rarely indicated during pregnancy, being primarily a personalized option. Regarding the postpartum period, it was observed that the absence of resolution/diminution of clinical symptoms after more than three months from birth is associated with worsening clinical symptoms or evolution to a chronic syndrome/disease. A decision on surgical treatment after childbirth becomes difficult in this context, being associated with a considerable possibility of spontaneous reversibility [7,45].

However, there are cases of significant impairment in functionality, both motor and sensory, and cases that are unresponsive or poorly responsive to available therapeutic measures. These advanced stages of the disease, when unresponsive, form a clinical picture dominated by physical dysfunction, which can become disabling for the mother. Often, the presence of permanent and high-intensity pain can generate a series of repercussions, such as anxiety, physical discomfort, and lack of rest. In these selected cases, considering the balance between the benefits for the mother and the fetus and the possible complications caused by lack of treatment, the justifiable advantage of surgical intervention should be discussed. Decompression techniques can be performed endoscopically or openly under local anesthesia. The literature data suggest the safety of this type of anesthesia for both the mother and the fetus [5,6,7,8,46]. In this context of incertitude, it is evident that the need to perform such surgical interventions only after informed consent is obtained is mandatory [47].

## 4. Conclusions

1. The high prevalence of pregnancy-onset CTS highlights the necessity of awareness of the increased possibility of developing this condition during pregnancy, to recognize PRCTS clinical symptoms, and to guide the patients towards physiotherapy treatment and correct antenatal care. All these measures, initiated and performed in a preventive and personalized manner, are meant to keep the woman’s quality of life either during or after pregnancy.
2. An early, preventive approach, including specific questionnaires, will allow the adoption of appropriate, personalized and targeted therapeutic measures. Initiating proper preventive and customized treatment will reduce the risk of specific related complications.
3. The management of PRCTS must include conservative educational measures that in cases of clinical symptoms’ persistence or worsening must be associated with the pharmacological treatment or surgical approach.
4. Even if PRCTS can evolve only as a transient neuropathy, the initiation of a preventive and curative personalized treatment must necessarily start from the very beginning by adopting conservative, non-invasive measures to avoid the development of irreversible nerve damage and the evolution of acute PRCTS into a chronic disease.
5. In PRCTS, making a therapeutic decision is challenging due to numerous factors, among which the most important are: (a) individual physiological status; (b) insufficient data regarding the side effects of some methods on pregnancy and lactation; and (c) possible postpartum reversibility of the clinical picture. In this context, the preventive and personalized approach remains the only way to achieve the best-possible results.

## Data Availability

No new data were created or analyzed in this study. Data sharing is not applicable to this article.
